# Supplementary material for: Tail engagement of arrestin at the glucagon receptor
Source: Nature. 2023 Aug 9;620(7975):904–10. doi: 10.1038/s41586-023-06420-x (PMC10447241; doi:10.1038/s41586-023-06420-x)
Supplement: Supplementary file 1 — Reporting Summary [file 41586_2023_6420_MOESM1_ESM.pdf]

nature portfolio

Corresponding author(s): Qiang Zhao and Beili Wu

Last updated by author(s): Jun 17, 2023

# Reporting Summary

Nature Portfolio wishes to improve the reproducibility of the work that we publish. This form provides structure for consistency and transparency in reporting. For further information on Nature Portfolio policies, see our Editorial Policies and the Editorial Policy Checklist.

## Statistics

For all statistical analyses, confirm that the following items are present in the figure legend, table legend, main text, or Methods section.

| n/a | Confirmed | |
|---|---|---|
| ☐ | ☒ | The exact sample size (*n*) for each experimental group/condition, given as a discrete number and unit of measurement |
| ☐ | ☒ | A statement on whether measurements were taken from distinct samples or whether the same sample was measured repeatedly |
| ☐ | ☒ | The statistical test(s) used AND whether they are one- or two-sided<br>*Only common tests should be described solely by name; describe more complex techniques in the Methods section.* |
| ☒ | ☐ | A description of all covariates tested |
| ☒ | ☐ | A description of any assumptions or corrections, such as tests of normality and adjustment for multiple comparisons |
| ☐ | ☒ | A full description of the statistical parameters including central tendency (e.g. means) or other basic estimates (e.g. regression coefficient) AND variation (e.g. standard deviation) or associated estimates of uncertainty (e.g. confidence intervals) |
| ☐ | ☒ | For null hypothesis testing, the test statistic (e.g. *F*, *t*, *r*) with confidence intervals, effect sizes, degrees of freedom and *P* value noted<br>*Give P values as exact values whenever suitable.* |
| ☒ | ☐ | For Bayesian analysis, information on the choice of priors and Markov chain Monte Carlo settings |
| ☒ | ☐ | For hierarchical and complex designs, identification of the appropriate level for tests and full reporting of outcomes |
| ☒ | ☐ | Estimates of effect sizes (e.g. Cohen's *d*, Pearson's *r*), indicating how they were calculated |

*Our web collection on statistics for biologists contains articles on many of the points above.*

## Software and code

Policy information about availability of computer code

| | |
|---|---|
| Data collection | Automated data collection on the Titan Krios was performed using serialEM 3.7. |
| Data analysis | The following softwares were used in cryo-EM data processing, model building, and structure validation: MotionCor2 v1.4.2, CryoSPARC 4.1, ResMap v1.1.4, ChimeraX v.1.1, COOT 0.8.9, PHENIX 1.19.2, and MolProbity 4.2.<br>The functional data were analyzed by GraphPad Prism 8.0.<br>The figures were prepared using PyMOL 1.8 and UCSF Chimera 1.15.<br>The sequence alignment graphic was prepared on the ESPript 3.0 server.<br>The flow cytometry data were collected and analyzed by GuavaSoft 3.1, Guava ExpressPlus panel. |

For manuscripts utilizing custom algorithms or software that are central to the research but not yet described in published literature, software must be made available to editors and reviewers. We strongly encourage code deposition in a community repository (e.g. GitHub). See the Nature Portfolio guidelines for submitting code & software for further information.

## Data

Policy information about availability of data

All manuscripts must include a data availability statement. This statement should provide the following information, where applicable:

- Accession codes, unique identifiers, or web links for publicly available datasets
- A description of any restrictions on data availability
- For clinical datasets or third party data, please ensure that the statement adheres to our policy

Atomic coordinates and cryo-EM density maps for the structures of GCGRV2RC–barr1 and glucagon–GCGRV2RC–barr1 complexes have been deposited in the Protein Data Bank (PDB) under identification codes 8JRU and 8JRV, respectively, and in the Electron Microscopy Data Bank under accession codes EMD-36606 and EMD-36607, respectively. The database used in this study includes PDB 4ZWJ, 5XEZ, 6LMK, 6U1N, 6UP7, 6TKO, 7R0C, and 7SRS.

## Research involving human participants, their data, or biological material

Policy information about studies with human participants or human data. See also policy information about sex, gender (identity/presentation), and sexual orientation and race, ethnicity and racism.

| | |
|---|---|
| Reporting on sex and gender | No human research participants are involved in this study. |
| Reporting on race, ethnicity, or other socially relevant groupings | No human research participants are involved in this study. |
| Population characteristics | No human research participants are involved in this study. |
| Recruitment | No human research participants are involved in this study. |
| Ethics oversight | No human research participants are involved in this study. |

Note that full information on the approval of the study protocol must also be provided in the manuscript.

# Field-specific reporting

Please select the one below that is the best fit for your research. If you are not sure, read the appropriate sections before making your selection.

☒ Life sciences ☐ Behavioural & social sciences ☐ Ecological, evolutionary & environmental sciences

For a reference copy of the document with all sections, see nature.com/documents/nr-reporting-summary-flat.pdf

# Life sciences study design

All studies must disclose on these points even when the disclosure is negative.

| | |
|---|---|
| Sample size | No statistical methods were used to predetermine sample size. All functional data were obtained from at least three independent experiments to ensure each data point was repeatable. Wild-type receptors were tested in parallel as controls with a large number of repeats. The sample sizes of the mutants were evaluated by calculating the standard error of the mean.<br>Sample size for the cryo-EM studies was determined by availability of microscope time and to ensure unambiguous modeling of most of residues that allowed us to obtain a high-resolution reconstruction. |
| Data exclusions | No data were excluded from the analyses. |
| Replication | The functional assays were performed in technical duplicate. All the independent experiments were performed within a month. All attempts at replication were successful.<br>The cryo-EM data were collected from two independent experiments performed within two months. |
| Randomization | Randomization is not relevant to this study, as all experiments did not allocate experimental groups. |
| Blinding | Blinding is not relevant to this study, as no subjective allocation was involved in any of the structural and functional experiments. |

# Reporting for specific materials, systems and methods

We require information from authors about some types of materials, experimental systems and methods used in many studies. Here, indicate whether each material, system or method listed is relevant to your study. If you are not sure if a list item applies to your research, read the appropriate section before selecting a response.

## Materials & experimental systems

| n/a | Involved in the study |
| --- | --- |
| ☐ | ☒ Antibodies |
| ☐ | ☒ Eukaryotic cell lines |
| ☒ | ☐ Palaeontology and archaeology |
| ☒ | ☐ Animals and other organisms |
| ☒ | ☐ Clinical data |
| ☒ | ☐ Dual use research of concern |
| ☒ | ☐ Plants |

## Methods

| n/a | Involved in the study |
| --- | --- |
| ☒ | ☐ ChIP-seq |
| ☐ | ☒ Flow cytometry |
| ☒ | ☐ MRI-based neuroimaging |

# Antibodies

Antibodies used: ANTI-FLAG M2-FITC antibody: Sigma, Cat#F4049, 1:120 diluted in TBS supplemented with 4% BSA and 20% viability staining solution 7-AAD (Invitrogen).

Validation: The ANTI-FLAG M2-FITC antibody was commercially obtained and the validation report is available in the supplier website: https://www.sigmaaldrich.cn/deepweb/assets/sigmaaldrich/product/documents/731/190/f4049dat-mk.pdf; https://www.sigmaaldrich.com/technical-documents/articles/biofiles/antibodies-to-peptides.html.

# Eukaryotic cell lines

Policy information about cell lines and Sex and Gender in Research

Cell line source(s): The Sf9 and HEK293F cell lines were originally obtained from Invitrogen.

Authentication: None of the cell lines have been authenticated.

Mycoplasma contamination: The cell lines were negative for mycoplasma contamination.

Commonly misidentified lines (See ICLAC register): No commonly misidentified cell lines were used.

# Flow Cytometry

## Plots

Confirm that:

☐ The axis labels state the marker and fluorochrome used (e.g. CD4-FITC).

☐ The axis scales are clearly visible. Include numbers along axes only for bottom left plot of group (a 'group' is an analysis of identical markers).

☐ All plots are contour plots with outliers or pseudocolor plots.

☐ A numerical value for number of cells or percentage (with statistics) is provided.

## Methodology

Sample preparation: Cell surface expression levels of GCGR and mutants were measured by incubating 10 ul cells with 15 ul monoclonal anti-Flag M2-FITC antibody (Sigma; 1:120 diluted in TBS supplemented with 4% BSA and 20% viability staining solution 7-AAD (Invitrogen)) at 4 °C for 20 min. After incubation, 175 ul TBS buffer was added and the fluorescent signal was measured using a flow cytometry reader (Guava easyCyte HT, Millipore).

Instrument: Guava easyCyte HT, Millipore

Software: The data were collected and analyzed by GuavaSoft 3.1, Guava ExpressPlus panel.

Cell population abundance: For each measurement, 2,000 cell events were collected and the fluorescence intensity of cell population with protein expression was calculated.

Gating strategy: Gating was determined by the Green-red fluorescence intensity to differentiate positive cells.

☐ Tick this box to confirm that a figure exemplifying the gating strategy is provided in the Supplementary Information.
